# Essential role of ABA signaling and related transcription factors in phenolic acid and lignin synthesis during muskmelon wound healing

**DOI:** 10.3389/fpls.2024.1404477

**Published:** 2024-05-21

**Authors:** Qihui Wang, Ning Liu, Ruirui Yang, Xuejiao Zhang, Ying Wang, Yongcai Li, Dov Prusky, Yang Bi, Ye Han

**Affiliations:** ^1^ College of Food Science and Engineering, Gansu Agricultural University, Lanzhou, China; ^2^ Department of Postharvest Science of Fresh Produce, Agricultural Research Organization, Rishon LeZion, Israel

**Keywords:** muskmelons, wound healing, abscisic acid, signal transduction, transcription factor, phenylpropanoid metabolism

## Abstract

Abscisic acid (ABA) is a key phytohormone involved in wound healing in fruits and vegetables, while fluridone (FLD) is its synthetic inhibitor. However, it is unknown whether ABA signaling and downstream transcription factors are involved in the synthesis of phenolic acids and lignin monomers in muskmelon wounds, and the underlying mechanisms. In our study, exogenous ABA promoted endogenous ABA synthesis by increasing the levels of β-carotenoid and zeaxanthin, activating 9-cis-epoxycarotenoid dioxygenase (NCED) and zeaxanthin epoxidase (ZEP), facilitated ABA signaling by increasing the expression levels of protein phosphatases type 2C (*CmPP2C*) and ABA-responsive element binding factors (*CmABF*), upregulated the expression levels of *CmMYB1* and *CmWRKY1*, and ABA induced phenylpropanoid metabolism by activating phenylalanine ammonia-lyase (PAL), 4-coenzyme A ligase (4CL), and cinnamyl alcohol dehydrogenase (CAD), which further increased the synthesis of phenolic acids and lignin monomers in muskmelon wounds during healing. Taken together, exogenous ABA induced phenylpropanoid metabolism and increased the synthesis of phenolic acid and lignin monomer in muskmelon wounds during healing, and may be involved in endogenous ABA synthesis and signaling and related transcription factors.

## 1 Introduction

Muskmelon (*Cucumis melo* L.) is capable of self-healing after wounding, which can effectively restrict water transpiration and prevent pathogenic infections by forming a sealing layer over wounds (Wang et al., 2021). Suberin polyphenolic (SPP) and lignin are the primary components of the closing layer of muskmelons (Xie et al., 2022). Through ester and ether linkages, hydroxycinnamic, ferulic, caffeic acid, and other phenolic acids polymerize to form SPP, and the ether linkages of *p*-coumaric, coniferyl, and sinapyl alcohols form lignin (Xie et al., 2022). Both SPP and lignin monomers are produced by phenylpropanoid metab *DkbZIP5*olism (Wang et al., 2019). PAL catalyzes the conversion of L-phenylalanine to trans-cinnamic acid. In a multistep reaction, cinnamic acid produces several phenolic acids, which are further converted by 4CL to the corresponding phenolic acid-CoA forms. CAD, a key enzyme in lignin biosynthesis, catalyzes the conversion of phenolic acid-CoA to *p*-coumaric, coniferyl, and sinapyl alcohols (Zhu et al., 2022).

Abscisic acid (ABA) is an essential phytohormone involved in both abiotic stress resistance and plant growth and development (Lim et al., 2015; Villalobos-González et al., 2016; Chen et al., 2020). ABA levels gradually increase during muskmelon development, reaching a peak within 42–56 days after flowering (Kojima et al., 2021). Endogenous ABA peaked before ethylene production, and the application of exogenous ABA promoted 1-aminocyclopropanecarboxylic acid (ACC) content and 1-aminocyclopropane-1-carboxylic acid oxidase (ACO) activity, thus facilitating the respiration and ethylene production of muskmelons, suggesting that ABA is involved in the ripening of muskmelons (Sun et al., 2013). Under cold stress, endogenous ABA levels were increased by upregulating the expression level of *CmNCED3*, which in turn activated the antioxidant system to enhance cold tolerance in muskmelon seedlings (Li et al., 2021). Additionally, ABA promotes wound healing in potato tubers, tomatoes, and kiwifruits (Han et al., 2018; Wei et al., 2020; Zhang et al., 2024).

Exogenous ABA plays a crucial role in stimulating both synthesis and signaling via endogenous ABA (Jia et al., 2022). First, zeaxanthin is converted to 9-cis-violaxanthin and 9-cis-neoxanthin via ZEP catalysis. The latter is oxidatively cleaved by NCED to produce xanthoxin. Xanthoxin is further metabolized by short-chain alcohol dehydrogenase/reductase (SDR) to abscisic aldehyde, which is then oxidized to ABA by abscisic aldehyde oxidases (AAO) (Chen et al., 2020). The synthesized ABA is then transduced through the PYR/PYL/RCAR-PP2C-SnRK2 core pathway, where PYR/PYL/RCAR receptors recognize ABA and PP2C serves as a critical regulator of ABA signaling. SnRK2, a kinase that modulates key ABA components, and downstream elements of ABA signaling, includes AREBs/ABFs (Hauser et al., 2011). Evidence indicates that exogenous ABA supplementation increases endogenous ABA levels by upregulating the expression of *ZEP* and *NCED*, which consequently increases the expression levels of *PacPP2C* and *PacSnRK2* to activate the ABA signaling pathway, thereby enhancing drought tolerance in cherry fruits (Shen et al., 2017). Similarly, exogenous ABA has been shown to augment endogenous ABA levels by upregulating *NCED* expression, which in turn increases *VvSnRK2.3* transcript levels, thereby inducing ABA signaling and enhancing grape fruit resistance to *Botrytis cinerea* (Zhang Y. T., et al., 2022). Exogenous ABA increases the levels of endogenous ABA in tomato fruit stem scars and enhances the content of suberin, which promotes tomato fruit wound healing (Leide et al., 2012). In addition, fluridone (FLD), an inhibitor of ABA biosynthesis, decreases zeaxanthin levels, an important substrate in ABA synthesis, by inhibiting lycopene β-cyclase (Wiczkowski et al., 2019).

As a critical element in downstream ABA signaling, *ABF* can recognize and bind downstream transcription factors, which then bind to the promoter regions of structural genes, including *PAL*, *CHS*, and *CHI*, to regulate their expression, and thereby activating phenylpropanoid metabolism (Hu et al., 2019). The ABA signaling cascade activates downstream *MYB* transcription factors, consequently upregulating the expression of structural genes, such as *PAL*, *4CL*, and *CAD*, thereby promoting the synthesis of phenolic acids, lignin monomers, and flavonoids in fruits. ABA application has been found to activate the expression of *Achn4CL* by upregulating *AchnMYB41* and *AchnMYB107*, thereby increasing the phenolic compound levels in kiwifruit (Wei et al., 2020). Additionally, exogenous ABA supplementation increases lignin content in apple fruit by facilitating the interaction between *MdSND1* and *MdMYB46*/*MdMYB83*, and subsequently upregulating the expression levels of *Md4CL*, *MdCCR*, and *MdCAD* (Chen et al., 2020). Furthermore, ABA increased the expression of *DkbZIP5*, which in turn activated *DkMYB4* transcript levels, increased the expression of *PAL* and *CHS*, and increased flavonoid content in persimmon fruits (Akagi et al., 2012).

ABA is an important phytohormone that is involved in wound healing in fruits and vegetables. However, whether ABA signaling and downstream transcription factors are involved in the synthesis of phenolic acids and lignin monomers in muskmelon wounds and the underlying mechanisms remain unknown. In this study, wounded muskmelons were treated with ABA and fluridone (FLD) to: 1) assess the levels of key substrates, the expression and activity of key enzymes involved in ABA synthesis, and endogenous ABA concentrations; 2) analyze the expression profiles of *CmPP2C*, *CmABF*, *CmMYB1*, and *CmWRKY1* (*CmPP2C*, *CmABF*, *CmMYB1*, and *CmWRKY1* were selected based on our previous transcriptomic results, and the expression level was upregulated by more than 6-fold as a basis for selection); and 3) evaluate gene expression patterns, key enzyme activities, and levels of phenylpropanoid metabolites. By investigating these aspects, we aimed to clarify the complex regulatory mechanisms of exogenous ABA in the synthesis of phenolic acids and lignin monomers during wound healing in muskmelon.

## 2 Materials and methods

### 2.1 Fruits, ABA, and FLD treatments

Muskmelons (*C. melo* L. cv. Yujinxiang) were harvested in July 2021, in Shuifu Town, Gaolan County, Gansu Province, China. At early maturity (hardness: 75.2 N, soluble solids content: 11%), fruits of consistent size, free from parasites, and mechanically undamaged were chosen, and eight fruits from each box were then packed in a cardboard box. They were taken to the laboratory and kept at room temperature (22 ± 2°C, RH 75%–80%).

ABA and FLD were obtained from Shanghai Yuanye Biotechnology Co., Ltd. (99% purity) and Sigma-Aldrich Corp. (98% purity), respectively.

### 2.2 Wounding and treatment of fruit

The fruits were rinsed, disinfected, and dried naturally, and then four artificial wounds were made around the fruit’s equator using a stainless steel peeler (Deng et al., 2023). The wounded fruits, including the wounds, were immersed in 0.5 mmol/L ABA (0.1% dimethylsulfoxide, co-solvent), 0.1 mmol/L FLD (0.1% dimethylsulfoxide, co-solvent), and distilled water (control) solutions to treat for 10 min, respectively, after which they were air dried and stored in a dark environment (22 ± 2°C, RH 75%–80%) for wound healing (Lulai et al., 2008). ABA and FLD concentrations were obtained from preliminary experimental screening, and 210 fruits were used for each treatment, with three replicates.

### 2.3 Sampling

At 0, 1, 3, 5, and 7 days after wounding, slices of 2 mm to 3 mm healing tissue were removed from the fruit wounds. After crushing, the samples were placed in centrifuge tubes and stored at −80°C (Xue et al., 2023).

### 2.4 Determination of β-carotenoid and zeaxanthin content

β-carotenoid and zeaxanthin content were determined by referring to Su et al. (2023). Samples (0.5 g) were extracted in the dark for 30 min using 5 mL of an extraction mixture (methanol/acetone/hexane = 1:1:2, v/v/v), and the supernatant was collected after centrifugation. After 1 h, the liquid portion was combined with 10% ethanol and potassium hydroxide and then rinsed twice. The extract was dehydrated using a centrifugal vacuum concentrator, mixed in 1 mL of a 1:1 v/v mixture of methyl tert-butyl ether and methanol, and finally filtered through a 0.22 µm microporous membrane. HPLC analysis was conducted using an Acclaim™ C30 column (250 mm × 4.6 mm, 5 µm) (Thermo Scientific, USA). The mobile phase consisted of methanol/methyl-tert-butyl ether (MTBE)/water (81/15/4, v/v/v) (A)-methanol (B) at a wavelength of 450 nm. The flow rate was 0.8 mL/min and the column temperature was 40°C. β-Carotenoid and zeaxanthin contents were expressed as mg kg^−1^ on a fresh weight basis.

### 2.5 Enzyme activities

The activities of ZEP and NCED were determined using an ELISA kit (Shanghai Enzyme-linked Biotechnology Co., Shanghai, China). The activities were expressed as U/mg, where U = 0.01 OD_450_ min^−1^.

PAL and 4CL were determined according to the method described by Wang et al. (2023), and CAD was measured according to the method described by Zhang X. J. et al. (2022). The above activities were quantified as U/mg prot, where U = 0.01 OD_290_ h^-1^, U = 0.01 OD_333_ min^-1^, and U = 0.01 OD_340_ min^-1^.

### 2.6 Endogenous ABA levels

After 20 min of homogenization of 1 g samples with 10 mL of a pre-cooled methanol:formic acid (99:1, v/v) solution, the samples were incubated at 4 °C for 12 h. After centrifugation, the supernatant was extracted, concentrated, and fixed in 1 mL. The HPLC-MC analysis was performed on an HPLC-MC (1260 Infinity II, Agilent, USA) equipped with a PorosheII 120, EC-C18 (4.6 mm × 150 mm, 4 m). The retention times of the ABA standards were compared to quantify the ABA sample. The concentration of ABA was determined using a standard curve. ABA levels were expressed as μg/g (Shin et al., 2021).

### 2.7 RT-qPCR analysis

Total RNA was extracted using an RNA simple Total RNA Kit (TIANGEN Biotech), and RNA extraction and cDNA synthesis were performed according to the method described by Wang et al. (2023). Based on our transcriptome data, the selected total genes were tested by qRT-PCR (unpublished results), and the 2^(−ΔΔCT)^ method to determine relative gene expression. Actin was used as a housekeeping gene and all primers are listed in **Table 1**.

**Table 1:** List of primer sequences used for RT-qPCR analysis.

Gene name	Gene ID	Forward Primer (5’-3’)
*CmZEP*	103497391	CAATGCCCTTGATGCTC CAAATAGGCTGTGAAACG
*CmNCED*	103503120	CGATGGGATGGTTCATG GTTCACCGATTGCCTTT
*CmPP2C*	103493532	CCTCTTGCCTGATTTGT CTCTGACTCGCCTGACC
*CmABF*	103495243	AGTTTGGCTCTTGGGATG GTTGCTGCTGCGGCTGA
*CmMYB1*	103495548	TACCTGGAAGAACGGATAA GTGCGTGGGTCACTGGA
*CmWRKY1*	103484390	TTTGTTGAATACATCCTCGTC ATCATCGTCCTCGCCTC
*CmPAL*	103491176	CTCATCCCGACCCACTC TCTCCATAACCCAATCAC
*Cm4CL*	103501216	ATGGAGTTACCGTGGCG GTCATTCCGTATCCCTGTC
*CmCAD*	103496188	GAGTGGGCTAAGAGGCG TCGGAGCTGACCAAGTAA
*CmActin*	103485254	GTGATGGTGTGAGTCACACTGTTC ACGACCAGCAAGGTCCAAAC

### 2.8 Protein content

The protein concentration of the enzyme solution was determined using the method by Bradford (1976). Bovine serum albumin (BSA) was used as the standard curve to calculate protein content.

### 2.9 Phenolic acids, lignin monomer, and lignin content

The concentrations of sinapic, caffeic, ferulic, *p*-coumaric, and cinnamic acids were determined at 325 nm, 325 nm, 322 nm, 310 nm, and 276 nm, respectively. The absorbance of coniferyl and cinnamyl alcohols was determined to be 263 nm and 273 nm, respectively. Phenolic acid and monomeric lignin contents were expressed in μg/g FW (Xie et al., 2022). The lignin content was determined according to Zheng et al. (2023) and expressed as OD_280_·g^−1^.

### 2.10 Statistical analysis

Each of the above measurements was performed in triplicate in biological replicates. Microsoft Excel 2022 was used to present the results as mean standard errors (SE), and multiple means for each treatment were compared using SPSS 25.0’s one-way ANOVA and the least significant difference (LSD) (*P <*0.05). Figures were generated using OriginLab OriginPro 8.5 (Northampton, USA) and tables were generated using Microsoft Word 2022.

## 3 Results

### 3.1 Exogenous ABA promotes endogenous ABA synthesis in fruit wounds

**Figure 1:**
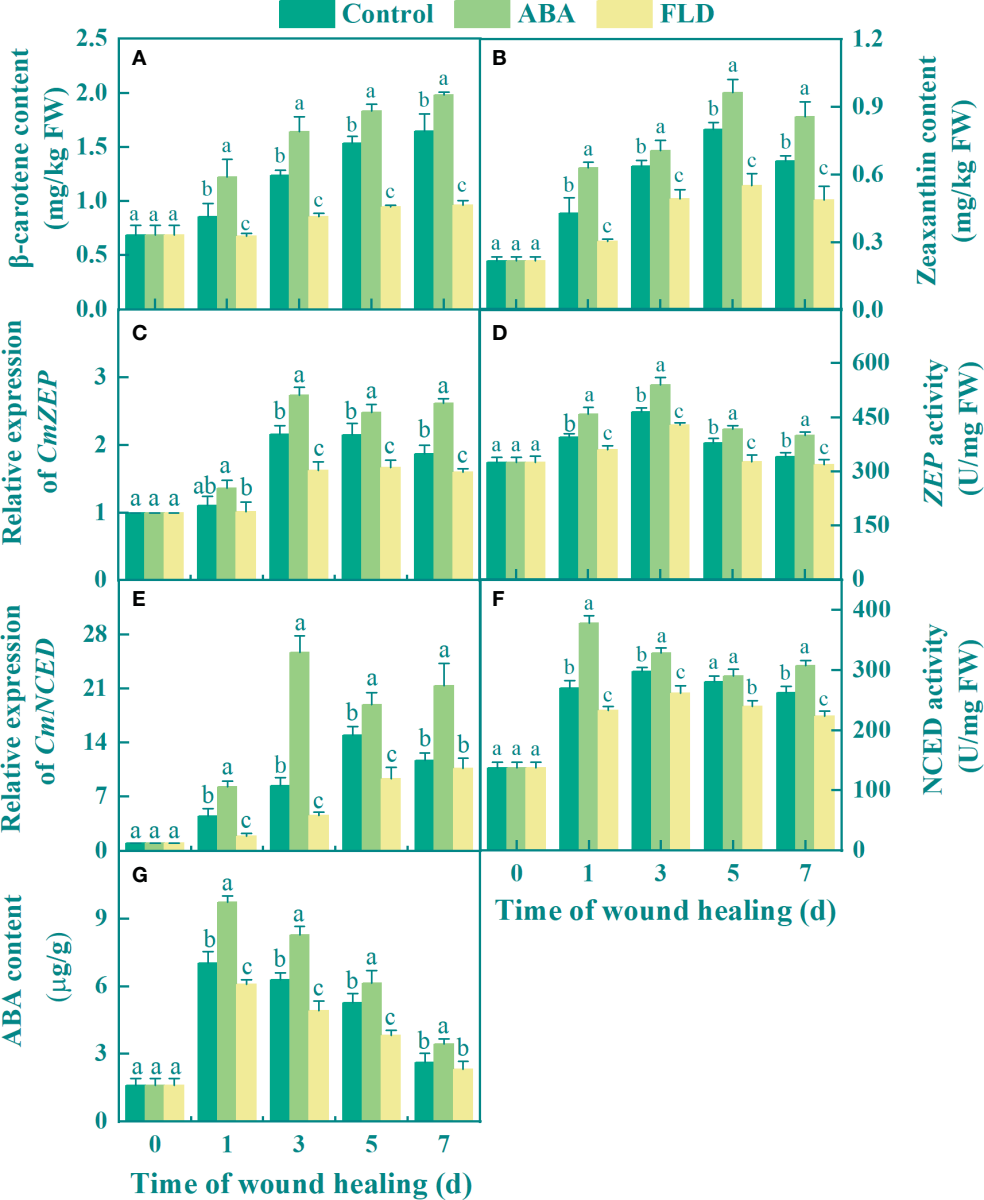
Effect of ABA and FLD treatment on the content of β-carotene **(A)** and zeaxanthin **(B)** the expression of *CmZEP* **(C)**, *CmNCED* **(E)**, the activity of ZEP **(D)** and NCED **(F)**, and ABA content **(G)** in muskmelon wounds during healing. Bars represent standard error ( ± SE). Different letters indicate significant differences (*P <*0.05).

During healing, the levels of β-carotene and zeaxanthin were significantly increased in the control and ABA treatment groups, whereas those in the FLD treatment group remained unchanged from 3 to 7 days. In comparison to the control, the ABA treatment group increased β-carotene and zeaxanthin levels by 20.17% and 29.72%, respectively, at 7 days (**Figures 1A, B**), but the FLD treatment group inhibited these levels by 46.61% and 26.69%, respectively (**Figures 1A, B**). In addition, the expression of *ZEP* and *CmNECD* in all the fruits increased over time, except for 1 day. The expression of *ZEP* was significantly different among the control, ABA, and FLD treatment groups. Except for day 7, the expression of *NCED* was significantly different among the ABA, control, and FLD treatment groups (**Figures 1C, E**). At 3 days, ABA-treated fruits had 1.26-fold and 3.04-fold higher levels of *CmZEP* and *CmNCED* expression, respectively, than the control. However, after 5 days, the FLD treatment group inhibited the expression of *CmZEP* and *CmNCED* to approximately 22.36% and 37.60%, respectively, compared to the control (**Figures 1C, E**). The activity of ZEP and NCED in all treatment groups showed a unimodal change during healing; the ABA treatment group showed significantly increased ZEP activity, which was inhibited by the FLD treatment (**Figures 1D, F**). Compared with the control, the activity of NCED in ABA application was increased by 39.78% at 1 day, while the FLD treatment group showed decreased activity (**Figures 1D, F**). Furthermore, 1 day after ABA treatment, endogenous ABA levels were 62% higher than in untreated fruits, whereas the FLD treatment group showed decreased endogenous ABA levels (27.32% lower at 5 days) (**Figure 1G**). The results showed that ABA application promoted endogenous ABA levels in muskmelon wounds by inducing *CmNCED* and *CmZEP* expression, whereas the FLD treatment group showed partial inhibition of ABA synthesis.

### 3.2 Exogenous ABA upregulates *CmPP2C* and *CmABF* expression in fruit wounds

**Figure 2:**
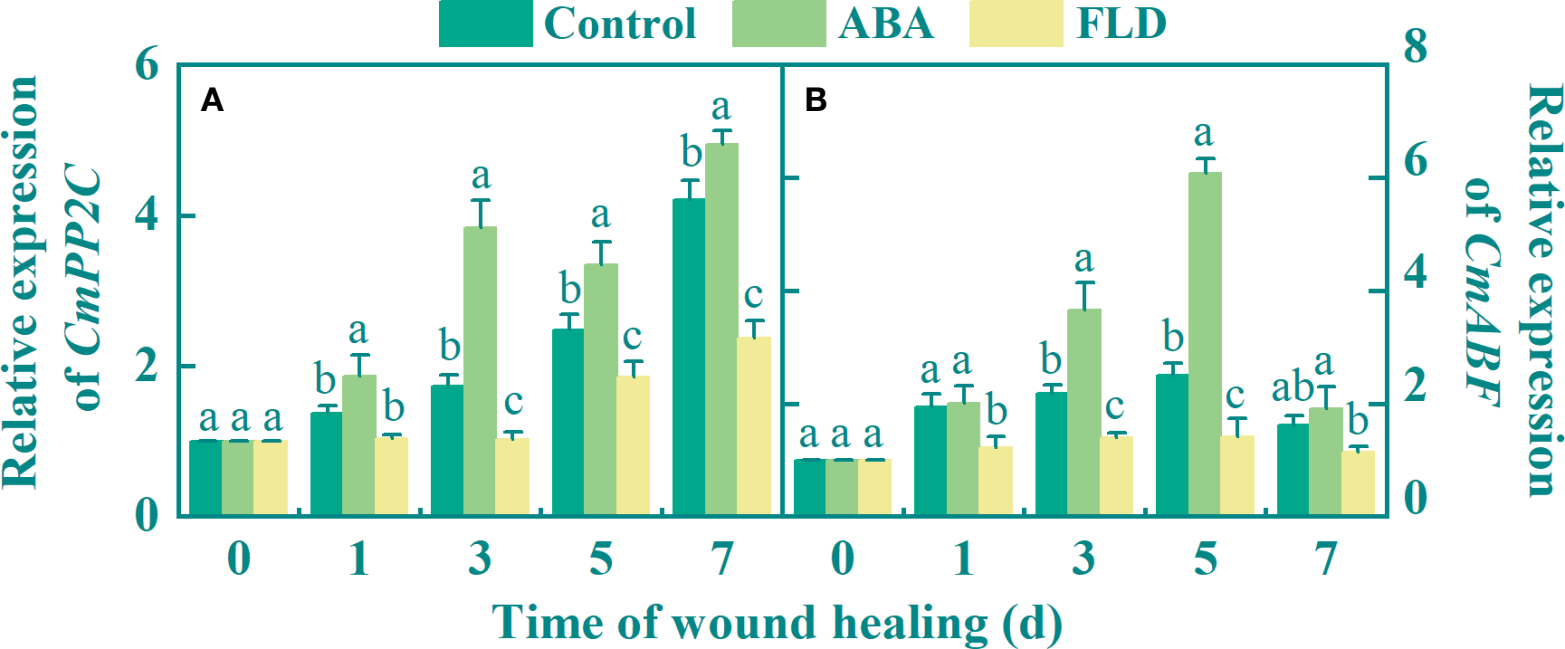
Effect of ABA and FLD treatment on the expression of *CmPP2C* **(A)** and *CmABF* **(B)** in muskmelon wounds during healing. Bars represent the standard error ( ± SE). Different letters indicate significant differences (*P <*0.05).

During healing, the expression of *CmPP2C* gradually increased over time. Compared to the untreated fruits, exogenous ABA upregulated *CmPP2C* expression (1.22-fold higher at 3 days). In contrast, at 7 days, the FLD treatment group showed reduced *CmPP2C* expression levels by 43.62% compared to the untreated fruit (**Figure 2A**). Except for days 1 and 7, there was a significant difference among the control, ABA, and FLD treatment groups. Five days after ABA treatment, *CmABF* expression was 1.42-fold higher than that in untreated fruits, whereas FLD application significantly reduced its expression by 43.64% (**Figure 2B**). These findings suggested that ABA treatment increased the levels of *CmPP2C* and *CmABF* expression and facilitated ABA signaling in muskmelon wounds, whereas the FLD treatment group showed partial inhibition.

### 3.3 Exogenous ABA upregulates *CmMYB1* and *CmWRKY1* expression in fruit wounds

**Figure 3:**
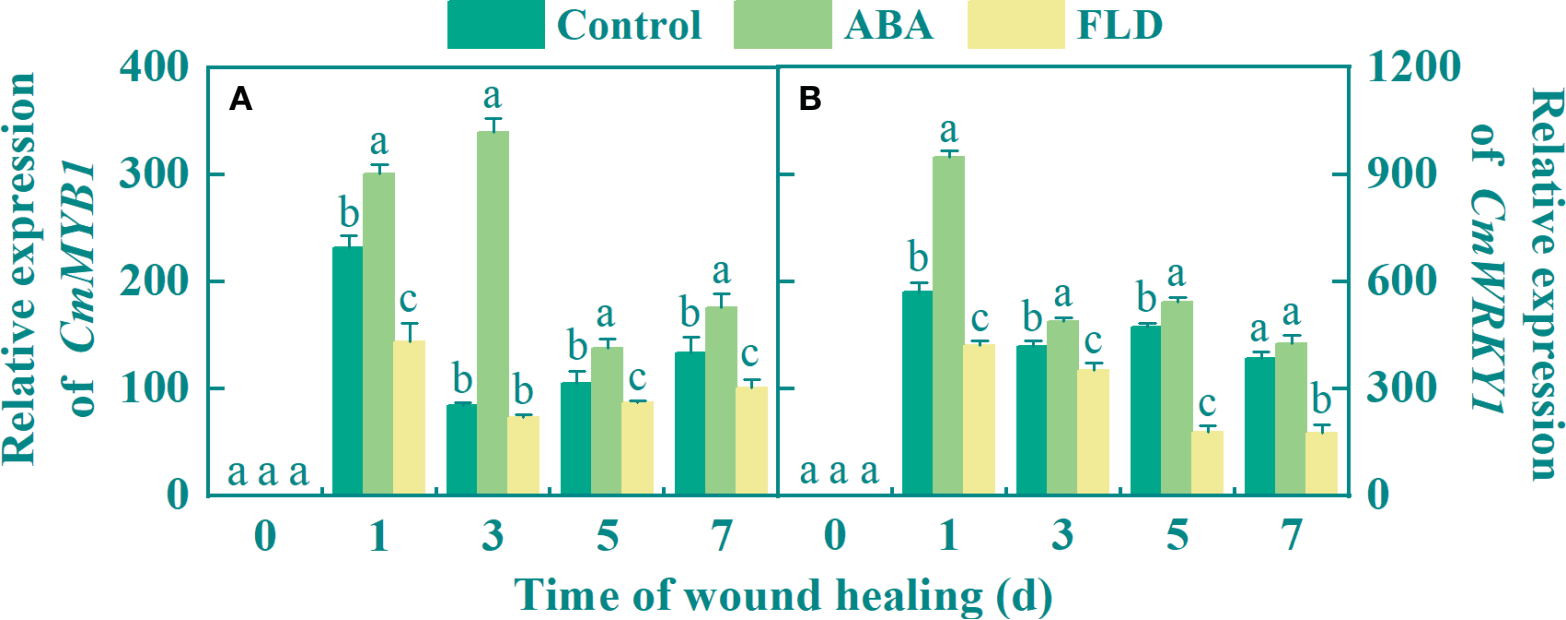
Effect of ABA and FLD treatment on the expression of *CmMYB1* **(A)** and *CmWRKY1* **(B)** in muskmelon wounds during healing. Bars represent the standard error ( ± SE). Different letters indicate significant differences (*P <*0.05).

The expression of *CmMYB1* in all fruits peaked during the early healing period. Except for day 3, there was a significant difference among the control, ABA, and FLD treatment groups. ABA application upregulated the *CmMYB1* transcript level by 4-fold at 3 days, but at 1 day after FLD treatment, *CmMYB1* expression was decreased by 37.92% compared with the control group (**Figure 3A**). Additionally, the expression of *CmWRKY1* gradually decreased in all fruits after peaking at 1 day, except at 7 days, where there were significant differences among the control, ABA, and FLD treatment groups (**Figure 3B**), At 1 day, ABA treatment had a higher *CmWRKY1* expression level, reaching 66.11% greater than the untreated fruits, but the FLD treatment group inhibited by 26.45% (**Figure 3B**). These findings suggested that ABA application induced *CmMYB1* and *CmWRKY1* transcriptional levels in wounds, while the FLD treatment group showed partial inhibition.

### 3.4 Exogenous ABA induces PAL, 4CL, and CAD in fruit wounds

**Figure 4:**
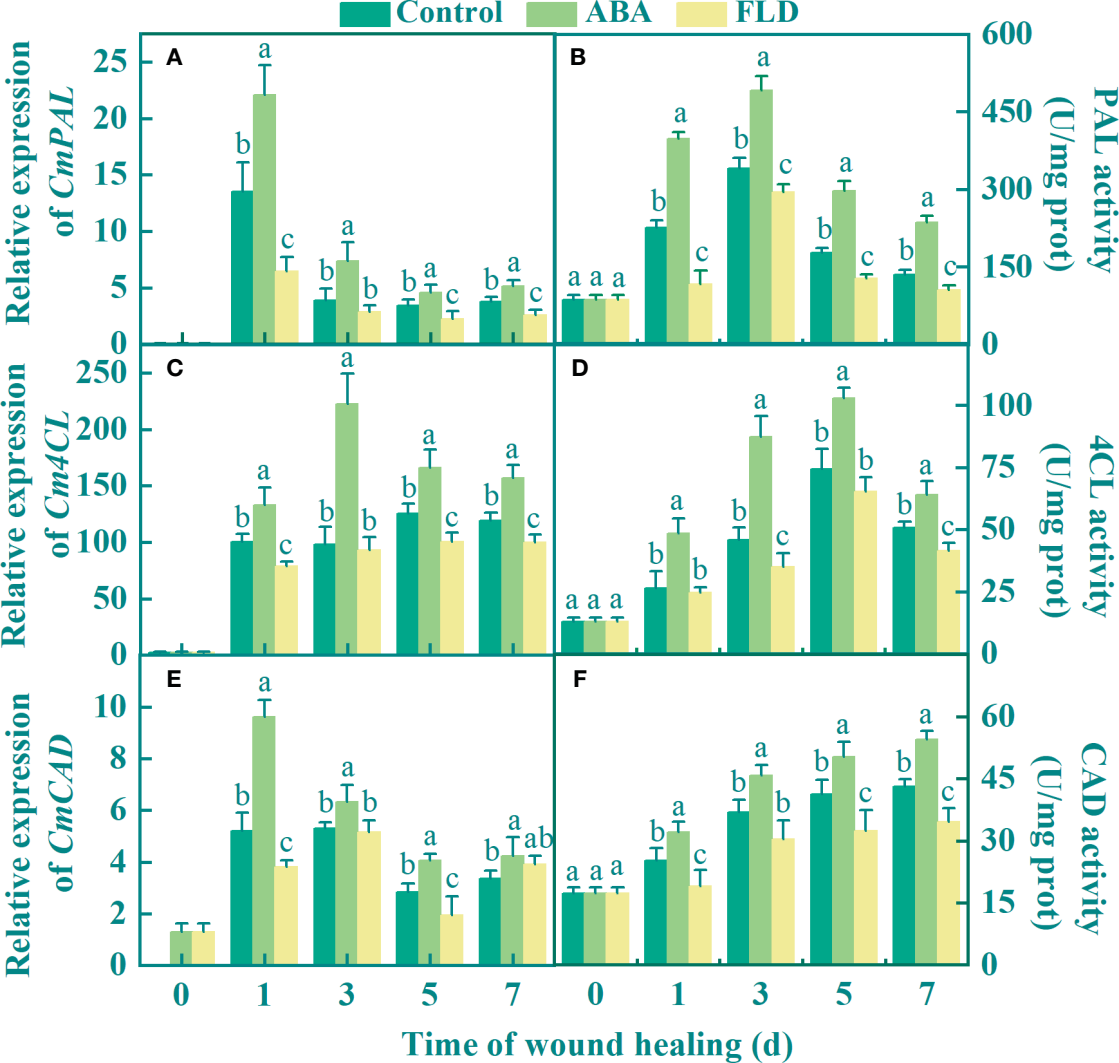
Effect of ABA and FLD treatment on the expression of *CmPAL* **(A)**, *Cm4CL* **(C)**, *CmCAD*, and **(E)** and the activity of PAL **(B)**, 4CL **(D)**, and CAD **(F)** in muskmelon wounds during healing. Bars represent the standard error ( ± SE). Different letters indicate significant differences (*P <*0.05).

The expression of *CmPAL* and *CmCAD* in all fruits peaked during the early healing period and then gradually decreased. Compared to the untreated fruits, the ABA treatment group upregulated the expression of *Cm4CL*, except at 3 days, and the FLD treatment group suppressed *Cm4CL* expression (**Figure 4C**). Compared with the control, ABA application upregulated the expressions of *CmPAL*, *Cm4CL*, and *CmCAD* by 63.40%, 32.86%, and 84.7%, respectively, after 1 day, whereas the FLD treatment group inhibited the expression levels of *CmPAL*, *Cm4CL*, and *CmCAD* (**Figures 4A, C, E**). PAL and 4CL activities in all fruits showed a unimodal pattern during healing. At 3 days, the PAL and 4CL activities in the ABA treatment group were higher than those in the control by 45.10% and 90.73%, respectively, whereas the activity in the FLD treatment group fruits was 9.16% and 23.81% lower than that of the control (**Figures 4B, D**). The activity of CAD gradually increased in all fruits, and there were significant differences between all groups, except for 3 days. At 7 days, CAD activity was 20.47% greater in the ABA treatment group than in the control, whereas it was 20.09% lower in the FLD treatment (**Figure 4F**). These findings demonstrated that ABA treatment induced PAL, 4CL, and CAD in wounds, whereas FLD treatment showed partial inhibition.

### 3.5 Exogenous ABA increased the levels of phenolic acids, lignin monomer, and lignin in fruit wounds

**Figure 5:**
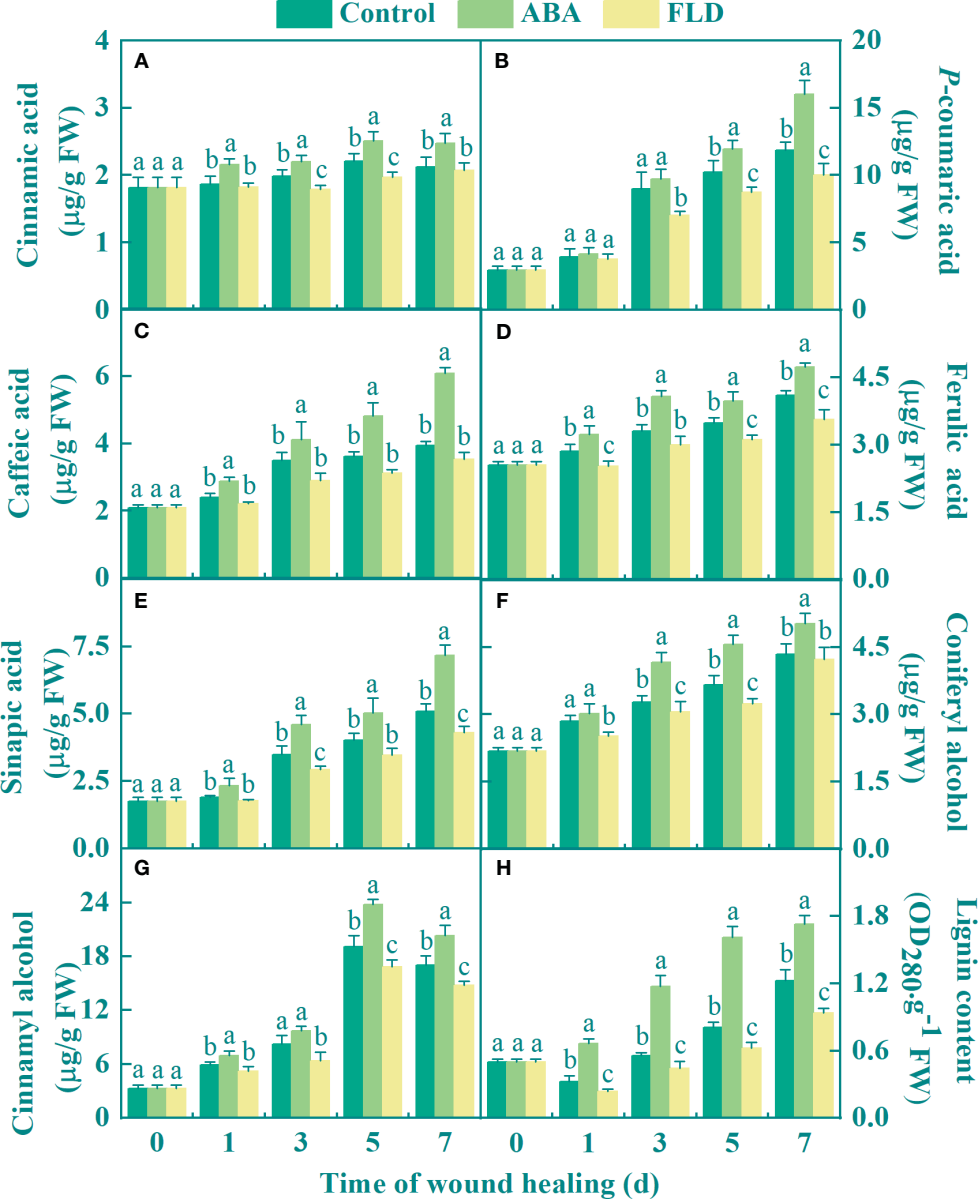
Effect of ABA and FLD treatment on the content of cinnamic acid **(A)**, *p*-coumaric acid **(B)**, caffeic acid **(C)**, ferulic acid **(D)**, sinapic acid **(E)**, coniferyl alcohol **(F)**, cinnamyl alcohol **(G)**, and lignin **(H)** in muskmelon wounds during healing. Bars represent the standard error ( ± SE). Different letters indicate significant differences (*P <*0.05).

The phenolic acid monomer, lignin monomer, and lignin contents gradually increased in all fruits during healing. The exogenous ABA treatment group showed increased the cinnamic acid content compared to that in the control group. However, except for days 1 and 7, FLD treatment group decreased the cinnamic acid content (**Figure 5A**). After 5 days, the ABA treatment group showed a higher *p*-coumaric acid content than the control, whereas FLD decreased its content after 3 days (**Figure 5B**). Seven days after ABA treatment, the caffeic acid level increased by 47.70% compared with that in the untreated fruits (**Figure 5C**). The ABA treatment group showed a 21.73% increase in ferulic acid levels at 3 days compared to the control, but the FLD treatment group a decrease in its content, except at 3 days (**Figure 5D**). Compared to the control, ABA treatment group also showed increased sinapic acid levels, but at 3 and 7 days, the FLD treatment group showed lower sinapic acid levels (**Figure 5E**). Furthermore, ABA treatment group increased the content of coniferyl and cinnamyl alcohols by 24.93% and 24.56%, respectively, compared with the untreated fruits at 5 days, respectively (**Figures 5F, G**). The FLD treatment group showed decreased coniferyl and cinnamyl alcohol content. After 7 days, ABA application enhanced the lignin level by 38.55%, in comparison to the control, while the FLD treatment group suppressed lignin content by 23.72% (**Figure 5H**). The results showed that ABA application promoted the production of phenolic acids (sinapic, caffeic, ferulic, *p*-coumaric, and cinnamic acids), lignin monomers (coniferyl and cinnamyl alcohols), and lignin content, whereas FLD showed partial inhibition.

## 4 Discussion

### 4.1 Exogenous ABA promotes endogenous ABA levels and signaling in fruit wounds

In this study, ABA treatment increased the levels of β-carotenoid and zeaxanthin, and activated ZEP and NCED, which increased the exogenous ABA concentration (**Figure 1**). This result is consistent with the finding that exogenous ABA treatment increases carotenoid content and upregulates the expression of *PpNCED1/2* and *PpZEP*, thereby promoting endogenous ABA levels in peach fruits (Tian et al., 2020). β-Carotene and zeaxanthin are key precursors in the ABA synthesis. ZEP and NCED are key enzymes in the ABA synthesis pathway. ZEP catalyzes the conversion of zeaxanthin into 9-cis violaxanthin and 9-cis neoxanthin. NCED oxidizes 9-cis neoxanthin to xanthoxin, which is further metabolized to abscisic aldehyde by SDR, and then oxidized to ABA by AAO (Chen et al., 2020). Therefore, we propose that exogenous ABA treatment increases the levels of β-carotenoids and zeaxanthin and activates the expression and activity of ZEP and NCED to promote endogenous ABA levels.

Furthermore, upregulation of *CmPP2C* and *CmABF* expression stimulated ABA signaling (**Figure 2**). Moreover, similar to the results of exogenous ABA in cherry fruit, the transcriptional levels of *PP2C1–3* were upregulated (Shen et al., 2017). ABA induces conformational changes in the conserved loop of the PYL protein structural domain, facilitating its interaction with PP2C to activate signaling pathways, ultimately leading to elevated transcription levels of *PP2C*, *ABF*, and other genes (Jia et al., 2022). Therefore, it is plausible that endogenous ABA induced by exogenous ABA triggers similar conformational changes in the conserved loop of the PYL protein, consequently activating ABA signaling.

Furthermore, FLD treatment inhibited β-carotenoid and zeaxanthin levels and suppressed ZEP and NCED activities, as well as exogenous ABA levels (**Figure 1**). In addition, FLD treatment reduced the transcript levels of *CmPP2C* and *CmABF* (**Figure 2**). FLD blocks lycopene biosynthesis by inhibiting phytoene desaturase (Góraj-Koniarska et al., 2017). In contrast, lycopene β-cyclase catalyzes the production of β-carotene and zeaxanthin using lycopene as a substrate (Wiczkowski et al., 2019). Therefore, we propose that FLD reduces β-carotene and zeaxanthin levels by blocking substrate production, which is an important precursor for ABA synthesis, leading to reduced endogenous ABA levels.

### 4.2 Exogenous ABA upregulates transcription factors and increases phenylpropanoid metabolites in fruit wounds

In the present study, exogenous ABA upregulated the expression of *CmABF*, *CmMYB1*, and *CmWRKY1*, and consequently activated PAL, 4CL, and CAD in wounds, thereby enhancing the accumulation of phenolic acids and lignin monomers (**Figure 5**). These results are consistent with those reported in persimmon fruit, where ABA induced upregulation of *DkbZIP5* and *DkMYB4* transcripts, consequently activating *PAL* and *CHS* expression (Akagi et al., 2012). The promoter region of *LcMYB1* contains an ABRE sequence that is recognized by ABA-induced *LcABF2/3*, which subsequently upregulates *CHS*, *CHI*, and *ANS* expression, thereby augmenting anthocyanin levels in lychee fruits (Hu et al., 2019). Moreover, *MYB* recognizes and binds to AC cis-acting elements in the *4CL* and *CAD* promoter regions, thereby enhancing the lignin content of pear fruit (Jia et al., 2018). Similarly, *WRKY* was shown to recognize and bind to W-box elements in the *PAL* and *4CL* promoter regions, thereby enhancing resistance to *Rhizopus stolonifera* in peaches (Ji et al., 2021). Furthermore, our bioinformatic analysis revealed the presence of transcription factor-binding sites for *MYB* or *WRKY* in the promoter regions of *CmPAL*, *Cm4CL*, and *CmCAD*. Therefore, we speculate that ABA signaling stimulated by exogenous ABA may induce transcription factors, such as *WRKY* or *MYB*, to recognize the promoter sequences of *PAL*, *4CL*, and *CAD*, thereby activating their expression levels and enhancing phenolic acid and lignin monomer accumulation. Exogenous ABA promotes ABA signaling by upregulating the expression levels of *CmPP2C* and *CmABF*, which play important roles in ABA signaling, and *CmABF* can activate downstream transcription factors. Exogenous ABA also upregulated the expression levels of *CmMYB1* and *CmWRKY1*, and the promoter regions of PAL, 4CL, and CAD contained binding sites for *MYB* and *WRKY*, suggesting that *CmMYB1* and *CmWRKY1* can bind and activate PAL, 4CL, and CAD to promote the synthesis of phenolic acids and lignin monomers. In addition, we found that ABA treatment reduced weight loss and disease index in muskmelon wounds (unpublished data), decreased water loss and pathogen invasion, and improved fruit quality during healing.

In our study, FLD treatment downregulated the expression of *CmPP2C*, *CmABF*, *CmMYB1*, and *CmWRKY1*; and inhibited *PAL*, *4CL*, and *CAD* in wounds; and reduced phenolic acid and lignin monomer levels. These results are partially comparable to the findings that FLD reduced PAL and 4CL expression levels in tomatoes (Han et al., 2018). FLD reduced anthocyanin accumulation in strawberry fruits by inhibiting *FaABI3* (the ABA response element) transcript levels and downregulating *FaMYB10* and *FaCHS* expression levels (Kadomura-Ishikawa et al., 2015). Therefore, we hypothesized that FLD could downregulate *ABF* transcript levels and inhibit the expression of *CmMYB1* and *CmWRKY1*, thereby reducing phenolic acid and lignin monomer levels and inhibiting wound healing in muskmelons.

Additionally, we postulated that the ABA signaling gene *CmABF* may upregulate the expression of *CmMYB* and *CmWRKY* by interacting with their promoter regions and subsequently binding to the promoter regions of *PAL*, *4CL*, and *CAD* to increase their expression levels, thereby enhancing the activity of key enzymes involved in phenylpropanoid metabolism and promoting the accumulation of phenolic acids and lignin monomers in muskmelon wounds. Further validation of this hypothesis can be pursued through transcriptional regulation studies in subsequent research efforts.

We also found an increase in jasmonic acid (JA) and salicylic acid (SA) levels in muskmelon wounds during healing (unpublished data), suggesting that JA and SA may be involved in muskmelon wound healing. It has been reported that increased JA levels upregulate the expression of *MdGAIPB* and *MdMYB108* and activate phenylpropanoid metabolism to accelerate wound healing in apples (Deng et al., 2023). SA enhanced the *in vitro* antioxidant capacity to maintain cell membrane integrity by modulating ROS homeostasis during the early healing of potato tuber wounds (Liu et al., 2024). Therefore, we believe that these hormones have similarities and may be involved in muskmelon healing by regulating the related metabolism.

## 5 Conclusion

**Figure 6:**
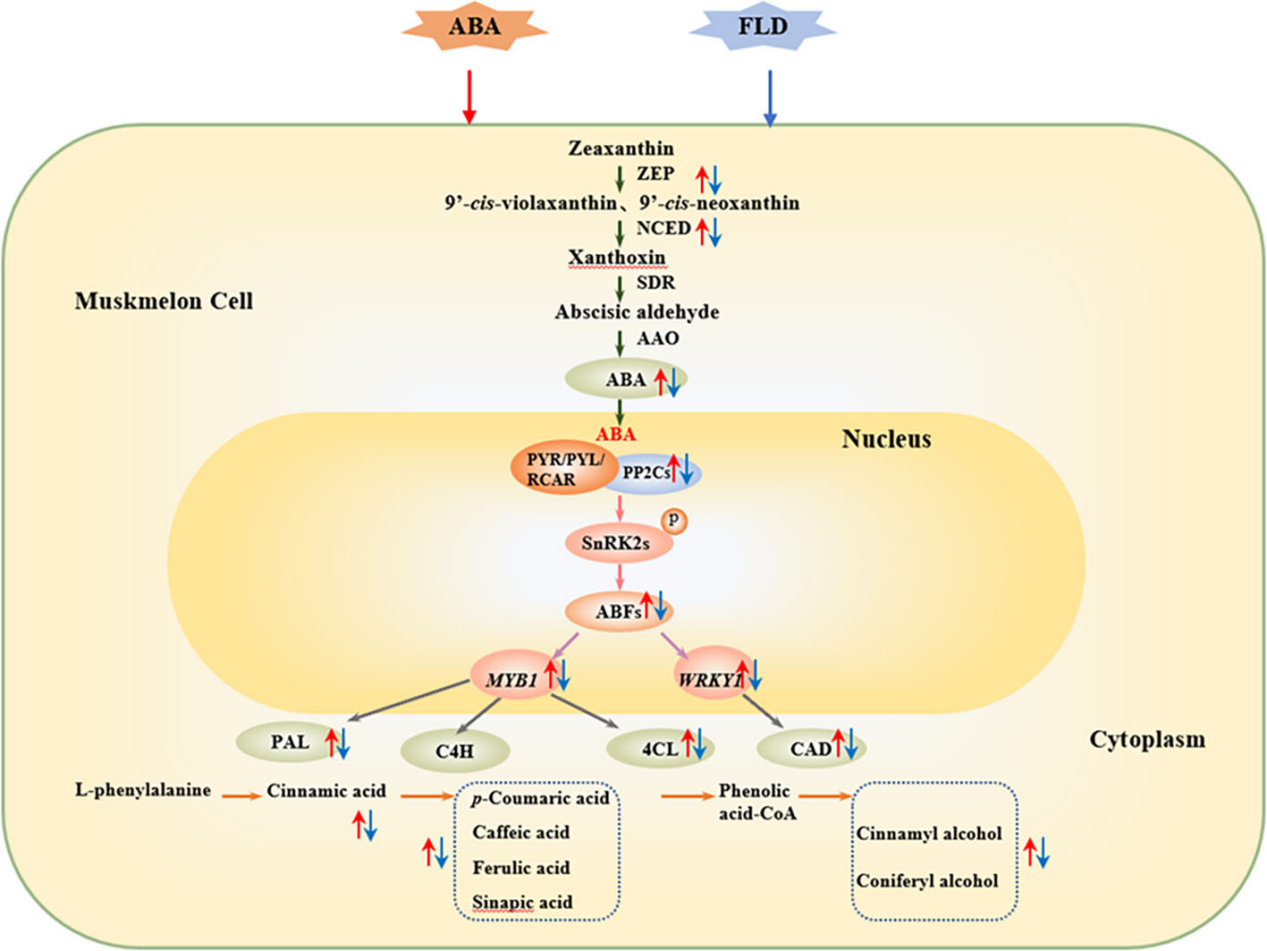
The possible pattern of ABA may promote the synthesis of phenolic acids and lignin monomers in wounds by activating downstream signaling elements and transcription factors. Green arrow represents the ABA synthesis pathway. Pink arrow represents the ABA signaling pathway. Purple arrow represents the interaction between the ABFs and transcription factors. Gray arrow represents transcription factors that activate the phenylpropanoid pathway. Orange arrow represents the phenylpropanoid metabolic pathway. Blue arrow represents SPP and lignin formation. Upward red arrow represents ABA-related level. Downward blue arrow represents FLD-related level. FLD, fluridone; ZEP, zeaxanthin epoxidase; NCED, 9-cis-epoxycarotenoid dioxygenase; ABA2, xanthoxin dehydrogenase; AAO, abscisic aldehyde oxidase; PYR, pyrabactin resistant; PP2C, protein phosphatases type 2C; SnRK2, Sucrose non-degrading protein phosphokinase; NADPH, nicotinamide adenine dinucleotide phosphate; SOD, superoxide dismutase; PAL, phenylalanine ammonia-lyase; C4H, cinnamate-4-hydroxylase; C3H, p-coumaroyl ester 3-hydroxylase; COMT, caffeic acid O-methyltransferase; 4CL, 4-coenzyme A ligase; CCoAOMT, caffeoyl CoA-O-methyltransferase; CAD, cinnamyl alcohol dehydrogenase.

In conclusion, exogenous ABA increases β-carotenoid and zeaxanthin levels, activates ZEP and NCED, and increases endogenous ABA levels. It enhances ABA signaling via the upregulation of *CmPP2C* and *CmABF*. In addition, ABA treatment upregulated *CmMYB1* and *CmWRKY1*, activated PAL, 4CL, and CAD enzymes, and leads to increased phenolic acids (sinapic, caffeic, ferulic, *p*-coumaric, and cinnamic acids), lignin monomers (coniferyl and cinnamyl alcohols), and lignin content in muskmelon wounds. These results suggest that ABA and related transcription factors play critical roles in regulating muskmelon wound healing. The possible patterns of ABA and related transcription factors play a critical role in regulating muskmelon wound healing (**Figure 6**).

## Data availability statement

The original contributions presented in the study are included in the article/**Supplementary Material**. Further inquiries can be directed to the corresponding authors.

## Author contributions

QW: Writing – original draft. NL: Methodology, Writing – review & editing. RY: Data curation, Visualization, Writing – review & editing. XZ: Formal analysis, Writing – review & editing. YW: Resources, Writing – review & editing. YL: Investigation, Writing – review & editing. DP: Conceptualization, Writing – review & editing. YB: Funding acquisition, Project administration, Supervision, Writing – review & editing. YH: Writing – review & editing.

## Supplementary material

Supplementary Figure 1 The accumulation of SPP **(A)** and lignin **(B)** in muskmelon wounds of CK、ABA and FLD treatments during healing.

Supplementary Figure 2 Analysis of cis acting elements in the *CmPAL* **(A)**, *Cm4CL* **(B)** and *CmCAD* **(C)** promoter regions.
