# Supplementary figures and images for: Essential role of ABA signaling and related transcription factors in phenolic acid and lignin synthesis during muskmelon wound healing
Source: Front Plant Sci. 2024 May 21;15:1404477. doi: 10.3389/fpls.2024.1404477 (PMC11149543; doi:10.3389/fpls.2024.1404477)

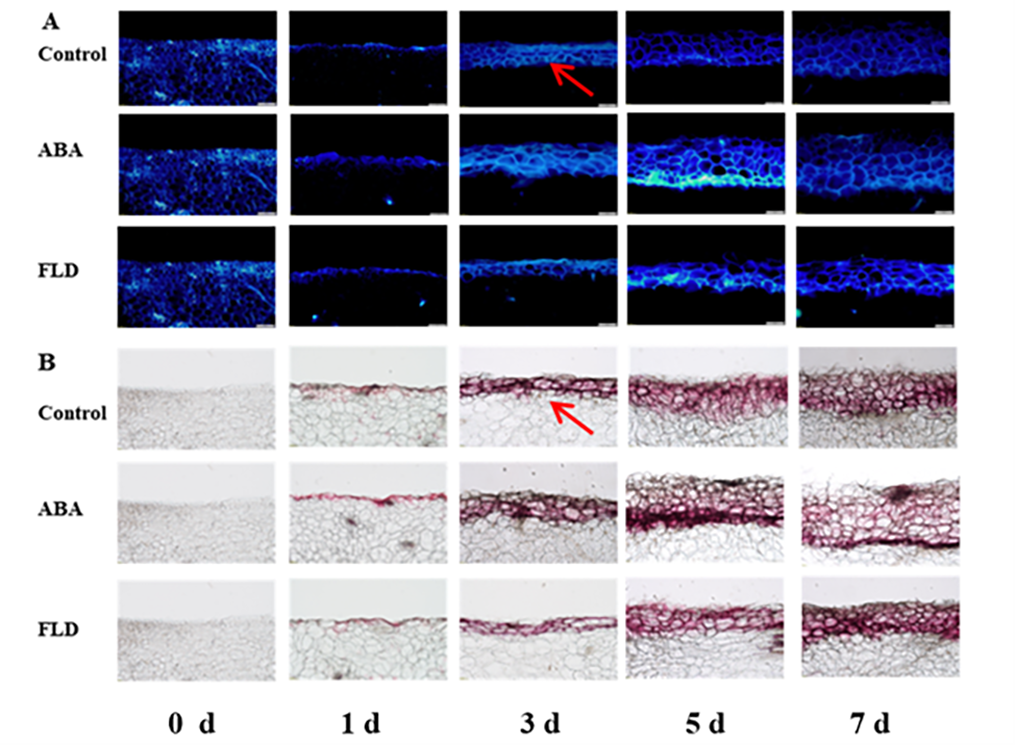

Supplement: Supplementary Figure 1 — The accumulation of SPP (A) and lignin (B) in muskmelon wounds of CK、ABA and FLD treatments during healing. [file Image_1.png]

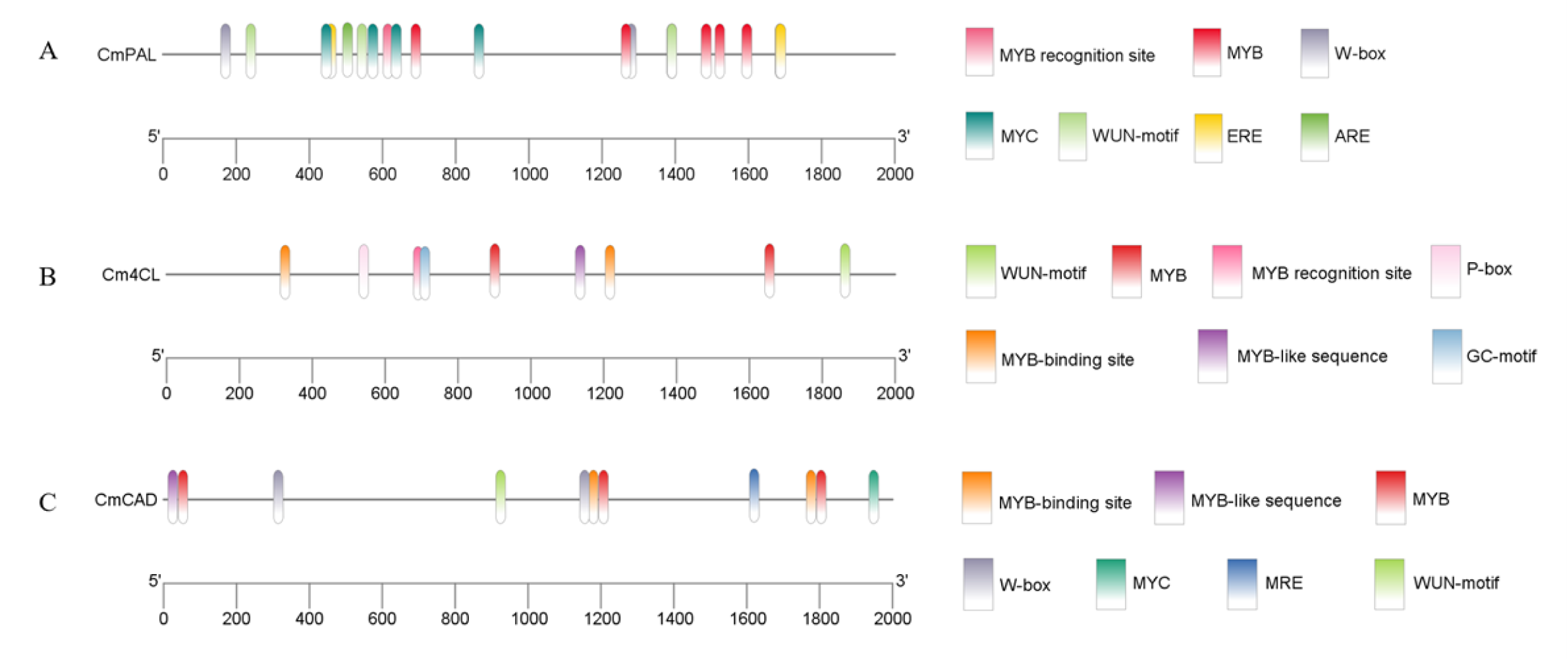

Supplement: Supplementary Figure 2 — Analysis of cis acting elements in the CmPAL (A), Cm4CL (B) and CmCAD (C) promoter regions. [file Image_2.png]
